# Facile assembly of upconversion nanoparticle-based micelles for active targeted dual-mode imaging in pancreatic cancer

**DOI:** 10.1186/s12951-018-0335-4

**Published:** 2018-01-29

**Authors:** Yong Han, Yanli An, Gang Jia, Xihui Wang, Chen He, Yinan Ding, Qiusha Tang

**Affiliations:** 1 Medical School of Southeast University, 87 Dingjiaqiao Road, Nanjing, 210009 China; 2 Affiliated Zhongda Hospital of Southeast University, Nanjing, 210009 China

**Keywords:** Nanoprobes, Pancreatic cancer, Molecular imaging, Upconversion nanoparticles

## Abstract

**Background:**

Pancreatic cancer remains the leading cause of cancer-related deaths, the existence of cancer stem cells and lack of highly efficient early detection may account for the poor survival rate. Gadolinium ion-doped upconversion nanoparticles (UCNPs) provide opportunities for combining fluorescent with magnetic resonance imaging, and they can improve the diagnostic efficacy of early pancreatic cancer. In addition, as one transmembrane glycoprotein overexpressed on the pancreatic cancer stem cells, CD326 may act as a promising target. In this study, we developed a facile strategy for developing anti-human CD326-grafted UCNPs-based micelles and performed the corresponding characterizations. After conducting in vitro and vivo toxicology experiments, we also examined the active targeting capability of the micelles upon dual-mode imaging in vivo.

**Results:**

We found that the micelles owned superior imaging properties and long-time stability based on multiple characterizations. By performing in vitro and vivo toxicology assay, the micelles had good biocompatibility. We observed more cellular uptake of the micelles with the help of anti-human CD326 grafted onto the micelles. Furthermore, we successfully concluded that CD326-conjugated micelles endowed promising active targeting ability by conducting dual-mode imaging in human pancreatic cancer xenograft mouse model.

**Conclusions:**

With good biocompatibility and excellent imaging properties of the micelles, our results uncover efficient active homing of those micelles after intravenous injection, and undoubtedly demonstrate the as-obtained micelles holds great potential for early pancreatic cancer diagnosis in the future and would pave the way for the following biomedical applications.

## Background

Pancreatic cancer, known as the emperor of cancer, is the most aggressive malignancies with a disappointing progression-free survival rate, causing countless deaths worldwide each year [1, 2]. Even for pancreatic cancer patients who has been through clinically curative resection, the 5-year survival rate is still below 5% due to local recurrence and widespread metastasis [3, 4]. Scientific researchers forecast that pancreatic cancer will ascend to the second leading cause of cancer death by 2030 [5]. Furthermore, patients with pancreatic cancer often suffer from debilitating symptoms, such as pain, early satiety, belching, jaundice and ascites due to pancreatic mass, thus leading to an unendurable quality of life. While surgical radical resection can be the best choice, many patients are pitifully not candidates for operative intervention due to late detection and progression of the disease. Therefore, early diagnosis of pancreatic cancer status would be extremely helpful to choose optimal therapy.

Fortunately, molecular imaging holds great promise for early pancreatic cancer based on detailed molecular and physiological information [6, 7]. Especially, upconversion nanoparticles (UCNPs) own some prominent advantages, such as weak background autofluorescence, deep tissue penetration, low photobleaching, and large anti-Stokes shifts, which are the prerequisite for in vitro or in vivo imaging [8–11]. Excitingly, with the integration of gadolinium ion (Gd^3+^) onto the nanoparticle core, dual-mode imaging probes can be designed with integrated magnetic and upconversion luminescence properties [12–14]. However, monodisperse UCNPs with highly uniform size and morphology are synthesized from organic systems. Therefore, subsequent optimal surface modification of UCNPs is necessary before in vivo applications. PEGylation, surface modification of UCNPs with polyethylene glycol (PEG), endow UCNPs with good biocompatibility, prolonged circulation time, and increased accumulation in tumor regions backed by the enhanced permeability and retention (EPR) effect [15, 16]. Unfortunately, many probes enable preferential accumulation in tumor sites through the EPR effect, which may not result in the desired imaging effects. Moreover, most nanoparticle approaches fall short of initial expectations when used against early stage of cancer, which is regarded as the key for potential curative therapy. This may be due to the inadequate accumulated of nanoparticles in EPR effect-lacking small clusters of disseminated tumor cells. Modification of the surface of nanoparticles using tumor-targeting moieties (folic acid, antibodies, and RGD, etc.) may hold potential to circumvent these hurdles [17–20]. Recently, epithelial cell adhesion molecule (EpCAM, also known as CD326) has captured the attention of researchers [21]. As one type of transmembrane glycoprotein highly expressed in most carcinoma cells, CD326 plays a critical role in tumor progression, invasion, and metastasis, which has been considered as one biomarker of cancer stem cells [22–24]. More importantly, previous studies show that CD326 plays a promise role in predicting patient’s prognosis and offering guidance for therapeutic strategy [25, 26]. As a result, CD326 may potentially serve as an effective tumor target for upconversion luminescence/magnetic resonance (UCL/MR) imaging simultaneously in early pancreatic cancer.

In this study, we developed a unique active targeting UCNP-based micelle (denoted as UPG) capable of dual-mode imaging and explored in vivo pancreatic cancer targeting behavior through intravenous injection. Such micelles can provide not only excellent T_1_ signal based on doped Gd^3+^, but also external lipid layer with satisfactory biocompatibility. Furthermore, as the integration of T_1_-weighted MR imaging contrast and fluorescence agents, the PEGylated UCNPs conjugated with anti-human CD326 antibody are potentially used for active targeting imaging due to specific antigen–antibody reaction. Therefore, our synthesized CD326-targeting micelles are validated for achieving substantially dual-mode MR/UCL imaging, which may signify a significant step forward in the biomedical field of cancer diagnostics.

## Methods

### Chemicals and agents

Yttrium(III) chloride hexahydrate (YCl_3_·6H_2_O; 99.9%), ytterbium(III) chloride hexahydrate (YbCl_3_·6H_2_O; 99.9%), erbium(III) chloride hexahydrate (ErCl_3_·6H_2_O; 99.9%), gadolinium chloride hexahydrate (GdCl_3_·6H_2_O; 99.999%), ammonium fluoride (NH_4_F), oleic acid (OA, 95%), 1-octadecene (ODE, 90%), 4, 6-diamidino-2-phenylindole dihydrochloride (DAPI) were purchased from Sigma-Aldrich (Australia). Methanol and sodium hydroxide (NaOH) were obtained from Lingfeng Chemical Reagent Company (Shanghai, China). MPEG-2000-DSPE (99.9%) and DSPE-PEG2000-Mal (99.9%) were purchased from Corden Pharma (Switzerland). Anti-human CD326 monoclonal antibody was purchased from eBioscience (Austria). Cell Counting Kit-8 was obtained from Dojindo (Tokyo, Japan).

### Synthesis of NaYF_4_:Yb,Er core nanoparticles

NaYF_4_:Yb (18%),Er (2%) core nanoparticles were synthesized using a thermal decomposition method with little modifications [27]. Typically, 1.6 mmol of YCl_3_·6H_2_O, 0.36 mmol of YbCl3·6H_2_O and 0.04 mmol of ErCl3·6H_2_O were added in a 100 mL three-neck round-bottomed reaction flask containing with 15 mL OA and 30 mL ODE. The resultant mixture was heated to 160 °C to form a light-yellow homogeneous solution under argon atmosphere. After the mixture was cooled down to room temperature, a methanol solution containing NH_4_F (0.3556 g) and NaOH (0.24 g) was slowly added, and the resulting solution was then kept stirring for 1 h. The solution was slowly heated to 100 °C under adequate argon flow for 0.5 h to completely remove methanol along with some water in the system. Subsequently, the reaction mixture was heated to 160 °C for 30 min with constant stirring under argon atmosphere. Then, the finally formed solution was heated to 300 °C for 1 h with vigorous stirring. Finally, nanoparticles were precipitated with ethanol and washed three cycles with cyclohexane/ethanol (1:3, v/v), and the resulting UCNPs were redispersed in 10 mL cyclohexane for further use.

### Synthesis of NaYF_4_:Yb,Er@NaGdF_4_ (UCNP@Gd) nanoparticles

The NaYF_4_:Yb,Er@NaGdF_4_ (UCNP@Gd) nanoparticles were synthesized using a seed-mediated method with some modifications [28]. Typically, 5 mL of cyclohexane solution of the above purified UCNPs was mixed with GdCl_3_·6H_2_O (0.12 mmol), 15 mL OA and 30 mL ODE in a 100 mL flask. The growth of the NaGdF_4_ shell and the following purification procedures for the UCNP@Gd particles were the same as those for NaYF_4_:Yb,Er nanoparticles. The purified nanoparticles were also redispersed in 10 mL cyclohexane or chloroform for further PEG modification experiments.

### Preparation of PEGylated UCNP@Gd (UPG) micelles

MPEG-2000-DSPE (23.22 mg) and DSPE-PEG2000-Mal (6 mg, MPEG-2000-DSPE: DSPE-PEG-mal = 4:1, molar ratio) were dispersed in 5 mL chloroform, then the above 5 mL chloroform solution of OA-capped UCNP@Gd nanoparticles was added in a 50 mL flask and dispersed uniformly after a period of sonication. Deionized water (5 mL) was then slowly introduced to the reaction system. After chloroform was thoroughly vaporized by facile evaporation under negative condition (65 °C, ~ 5 min), the micelles became water soluble. The additional micelles without UCNP@Gd were removed via centrifugation at 5000×*g*, and the large aggregates were discarded. The products were dispersed in water under room temperature.

### Construction of UCNP@Gd@PEG-CD326 micelles

UCNP@Gd@PEG-CD326 (UPG-CD326) micelles were prepared as described previously with slight modifications. Briefly, sulfhydryl group was conjugated to anti-human CD326 monoclonal antibody (20 μg) by incubating with 90 μg of Traut’s reagent in 0.5 mL phosphate buffer saline (PBS, 0.1 M, pH = 9.0). Anti-human CD326-SH was coincubated with the above UPG micelles in 4 mL PBS (0.1 M, pH = 8.0) at 4 °C overnight. The final anti-human CD326-grafted UPG micelles (UPG-CD326) were collected by centrifugation several times, and lastly dispersed in 5 mL deionized water for further use.

### Characterization of the synthesized nanoparticles

The morphology of the as-synthesized nanoparticles was examined through a transmission electron microscopy (TEM, JEOL JEM-1200EX microscope, Japan). The size distribution of UCNP-based micelles was analyzed by software Image J. As for the morphology analysis of micelles, pre-diluted solutions of micelles (dispersed in water) were deposited on a copper grid/carbon film for 5 min. Afterwards, one drop of staining solution (2% phosphotungstic acid) was added and allowed to contact for 10 s before TEM analysis. The Gd^3+^ concentration of samples were established by inductively coupled plasma atomic optical emission spectrometer (ICP-OES). The emission and excitation spectra were acquired using an F-2700 Fluorescence Spectrophotometer (Hitachi, Japan) equipped with an external 980 nm continuous-wave near infrared (NIR) laser (NIR-LDR-980-10 W, Xilong Co., Shanghai, China) with maximum power of ~ 10 W. The infrared spectra were inspected with a Fourier transform infrared (FTIR) spectrometer (Nicolet Co., USA). The DLS measurements was explored using a Zeta-Plus analyzer (Brookhaven Instruments Co., Holtsville, USA).

### T_1_-weighted MR imaging in vitro

MR imaging experiment in vitro was conducted on a clinical 7.0 T MR scanner (Pharma Scan, Brukers, Germany). T_1_ relaxation times were ascertained with a multi-echo spin-echo sequence (16 echoes; repetition time (TR) = 2500 ms; echo time (TE) = 22–352 ms). For each sample, T_1_-weighted MR images of seven different concentrations of Gd^3+^ (0, 4.29, 8.58, 17.2, 34.3, 68.7, 137.4 μmol/mL) were analyzed. The relaxivity coefficient (R_1_) was acquired as the gradient of the plot of R_1_ (R_1_ = 1/T_1_) versus the molarity of magnetic atoms.

### Cytotoxicity evaluation of the micelles

RAW 264.7 macrophages were chosen as to evaluate the in vitro cytotoxicity of the as-prepared micelles. Generally, the cells were coincubated with UPG micelles of different concentrations (3–1000 μg/mL) in each well of a 96-well plate with 3 × 10^3^ cells/well (4-wells per group) for 24 h and 48 h, respectively. Meanwhile, the treated cells were further incubated with 150 μL fresh medium containing 10 μL Cell Counting Kit-8 (CCK-8) solutions for additional 4 h. Then the absorbance of each well was recorded by a microplate reader at the wavelength of 450 nm. Cell viability was finally expressed as the percentage of viable cells in contrast to untreated control cells.

### Cellular uptake study using confocal laser scanning microscopy (CLSM)

Human pancreatic cancer cell line BxPc-3 (2 × 10^3^ cells/well, CD326 expression: 80.5%) was seeded into 96-well plates to allow a firm adherence. After incubation for 24 h, cells were then treated with UPG-CD326 (i.e., targeted micelles) and nontargeted UPG micelles (i.e., without anti-human CD326 conjugation) at a micelle concentration of 200 µg/mL. The blocking experiment with the coincubation of free anti-human CD326 (3 × 10^−6^ M) and targeted micelles was also conducted. Afterward, the cells were rinsed with PBS trice to remove the unlabeled nanoparticles. Subsequently, the cells were further incubated with 2 μg/mL DAPI for nuclei staining. The samples were then subjected to a CLSM with an external 980 nm light source.

### Toxicology of the micelles in vivo

Healthy athymic balb/c female nude mice (body weight: 22 ± 2 g, 4 weeks old) were purchased from the Model Animal Research Center of Nanjing University and raised at Laboratory Animal Center, medical school of Southeast University. All animal procedures were in accordance with the Guidelines of animal care committee of Southeast University. Then 18 mice were intravenously treated with three groups (six mice per group) including: (1) control group: 150 µL saline; (2) targeted group: UPG-CD326 micelles in physiological saline (25 mg/mL, 150 µL); (3) non-targeted group: UPG micelles in physiological saline (25 mg/mL, 150 µL). The mice were anesthetized and the blood samples were acquired in 7 days of post-injection for the following biochemistry assays.

### Subcutaneous mouse model of human pancreatic cancer

Twelve healthy athymic balb/c female nude mice (4 weeks old with average weight of 20 g) were obtained from the Model Animal Research Center of Nanjing University. To establish the experimental model of human pancreatic cancer, inoculation with 2 × 10^6^ BxPc-3 cells was completed by subcutaneous injection into the right axilla of mice. When the tumors reached 5–6 mm in diameter, the mice were randomly divided into two groups (n = 6 in each group). The mice were intravenously injected with 150 µL of UPG/UPG-CD326 micelles in physiological saline at a micelle concentration of 16 mg/mL.

### UCL imaging in vivo

The UCL images of tumors were acquired with the Maestro in vivo optical imaging system (excitation: 980 nm, emission: 650 nm, exposure time: 400 ms; Maestro EX, Cri, USA) at different time-points including pre-injection, 4, 8, 24 and 48 h post-injection and analyzed by the Maestro 2.10.0 software (Maestro EX, Cri, USA). A 10 W adjustable CW NIR laser (λ = 980 nm) was used as an excitation source with a beam size of 30 mm, larger than the size of the tumor. The power density of excitation light was 320 mW/cm^2^. The distance between the skin on top of the tumor and the laser probe was about 50 mm.

### MR imaging in vivo

In vivo MR imaging test was conducted at a 7.0 T Micro-MRI instrument (PharmaScan, Brukers, Germany). Mice were initially anesthetized with a 4% isoflurane/air mixture delivered via a gas hood and kept body temperature at 37 °C. The MR image and the corresponding MRI signal intensity were obtained at the predetermined time-points including pre-injection, 4, 8, 24 and 48 h post-injection (16 mg/mL, 150 µL), respectively.

### Biodistribution and targeting efficiency of the micelles

Balb/c nude female mice were injected with UPG-CD326 and UPG micelles (16 mg/mL, 150 µL per mouse) via tail vein, respectively. Five mice from each group were euthanized and sacrificed at various time points including 12, 24, 48 post-injection, and tumor tissues and major organs (heart, liver, spleen, lung, kidney, and pancreas) were collected. Afterwards, they were indigested with HNO_3_ and HCl (v/v = 1/3), then heated at 70 °C for 5 min. The samples were diluted with ultrapure water to a 5% acid solution and then prepared for Y element by inductively coupled plasma mass spectrometry (ICP-MS) analysis. Besides, the targeting efficiency of the micelles at different time points were also calculated. Targeting efficiency (%) = (the total Y element content in the tumor/the total Y element content in the injected micelles) * 100%.

### Cellular localization of the micelles by bio-TEM observation

To verify the cellular localization of the micelles, the tumor tissues were surgically removed from the sacrificed BxPc-3 tumor-bearing mice, and fixed with 2.5% glutaraldehyde instantly overnight. The tissues were then cut into small pieces of ~ 1 mm^3^. Successively, tissue samples were stained 1% uranyl acetate overnight in the dark, and then dehydrated by ethanol alcohol with different concentrations (from 25 to 100%). Finally, the samples were embedded in resin, and then cured in an oven 60 °C (2 days) for the following TEM analysis.

### Statistical analysis

All of the obtained values are expressed as mean ± standard deviation (SD). The data were analyzed using the SPSS 20.0 software. All experiments were performed in triplicates. A *p* value of 0.05 was considered statistically significant.

## Results and discussion

### Synthesis and characterization of UCNP-based micelles

**Fig. 1:**
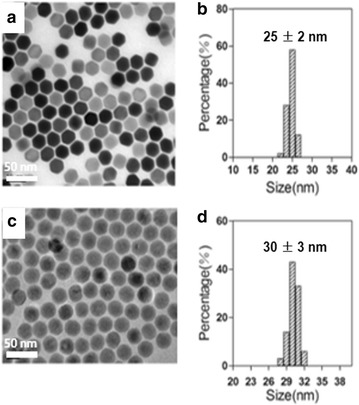
TEM images and size histograms of NaYF_4_:Yb,Er core nanoparticles (**a**, **b**) and NaYF_4_: Yb,Er@NaGdF_4_ core@shell nanoparticles (**c**, **d**)

**Fig. 2:**
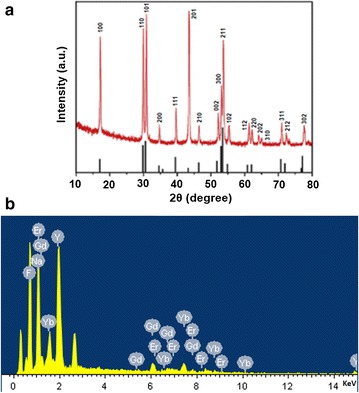
**a** X-ray powder Diffraction (XRD) and **b** emission diffraction X-ray (EDX) spectrum of NaYF_4_:Yb,Er@NaGdF_4_ core@shell nanoparticles

The NaYF_4_:Yb,Er core UCNPs were synthesized using a thermal decomposition method. As shown in Fig. 1a, TEM image indicated that these nanoparticles were monodisperse with a narrow size distribution (Fig. 1b, diameter: 25 ± 2 nm). Doping Gd^3+^ into UCNPs has been demonstrated to be a facile way for realizing enhanced MR/UCL imaging for tumors. Given this knowledge, the core@shell structured NaYF_4_:Yb,Er@NaGdF_4_ (UCNP@Gd) particles were constructed according to a previous report, and we find that these nanoparticles kept their spheric particle morphology and favorable dispersibility (Fig. 1c, d) and possessed an average diameter of 30 ± 3 nm after the shell growth under TEM observation. In addition, X-ray powder diffraction (XRD) patterns (Fig. 2a) confirmed the uniform hexagonal structure of UCNP@Gd nanoparticles (JCPDS no.28-1192), and this is favorable for achieving highly luminescent rare-earth particles. Furthermore, energy-dispersive X-ray (EDX) spectroscopy demonstrated that Gd^3+^ was successfully doped into the surface of UCNPs (Fig. 2b), which is the prerequisite for the following UCL/MR imaging.

**Fig. 3:**
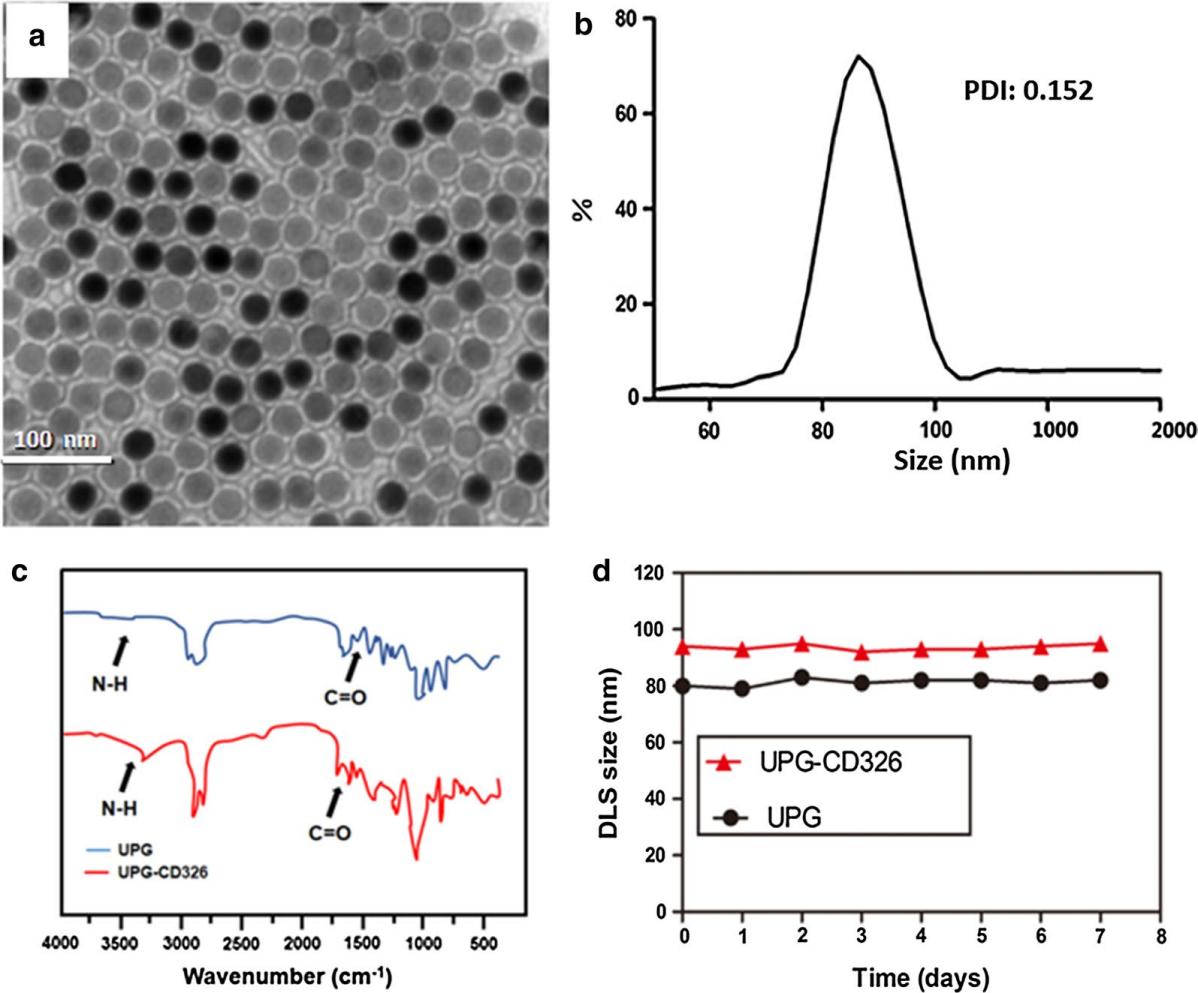
TEM image (**a**) and dynamic light scattering (DLS) measurement (**b**) of UPGs micelles. **c** Fourier transform infrared (FTIR) spectra of UPGs (blue line) and UPGs-CD326 (red line). **d** DLS size measurements of UPG and UPG-CD326 dispersed in PBS for varied time durations (0–7 day)

It is known that appropriate surface modification is critical for UCNPs to apply in biomedical field. To ameliorate their biocompatibility and stability in internal environments, we adapted the strategy of PEGylation to modify UCNPs. A thin PEG layer was encapsulated onto the core–shell UCNP@Gd with the aid of a rotation-evaporation method, thus the UCNP-based micelles (UPG) were prepared and dispersed in water under room temperature. After negative staining with 2% phosphotungstic acid, a thin and uniform coating was clearly visible on the UPG micelles (the average size was 85 ± 4 nm) in the correlative TEM image (Fig. 3a, b), demonstrating their uniformity in shape and satisfying surface modification without apparent agglomeration (the polydispersity index is 0.152).

Subsequently, anti-human CD326 antibody was covalently grafted onto the micelles based on an esterification reaction under the catalysis of Traut’s reagent, as shown by the corresponding FTIR characterization (Fig. 3c). The spectrum of UPG micelles showed weak N–H peak around 3600 and 3200/cm and weak C=O in 1674/cm, respectively. Meanwhile, both of the two peaks increased in the spectrum of UPG-CD326, which indicated the successful combination of anti-human CD326 monoclonal antibody to UPG micelles. Moreover, the DLS results showed that both of the micelles were well dispersed in PBS for at least 1 week, thus displaying their relatively high colloidal stability (Fig. 3d), and these properties meet the requirements of biomedical administrations.

### Optical and magnetic resonance properties of the micelles

**Fig. 4:**
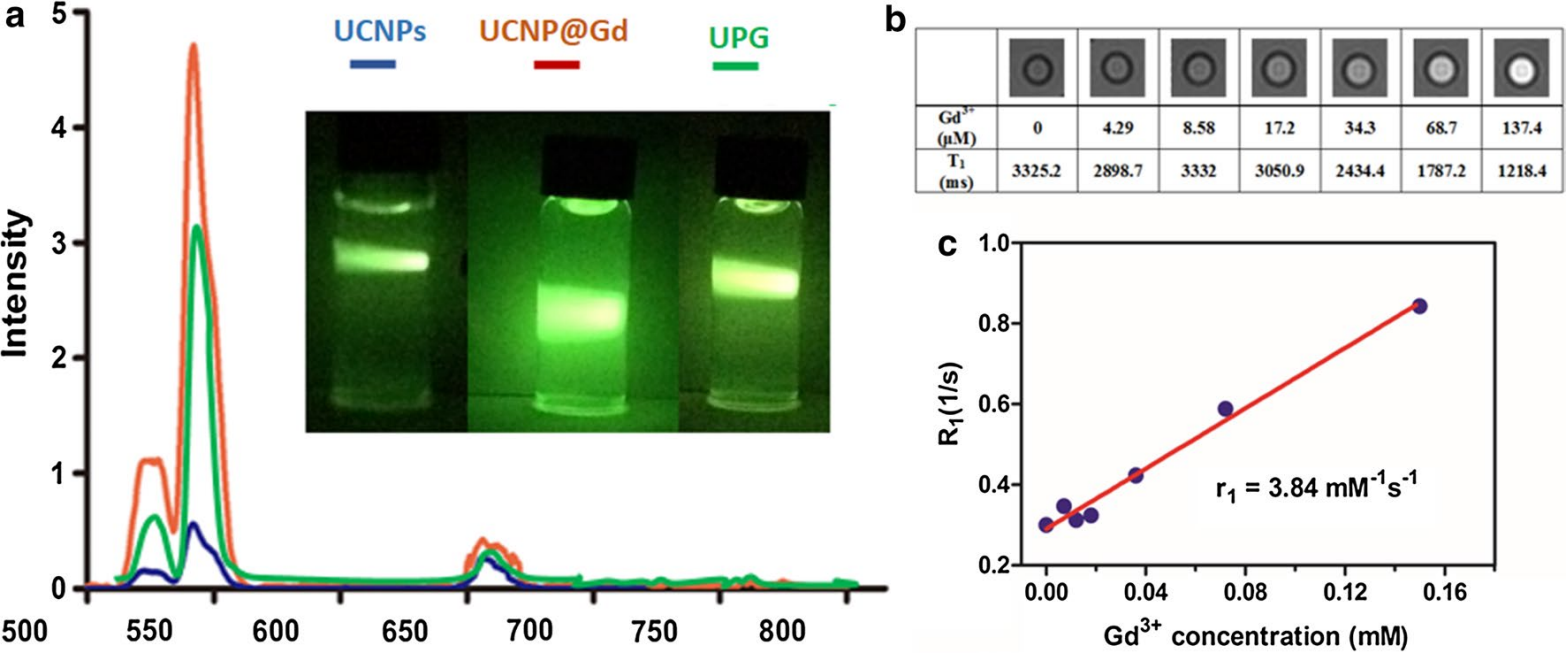
Normalized upconversion luminescence spectra of core, core@shell particles in cyclohexane and UPG micelles in PBS under 980 nm excitation (**a**). The insets are full-color photographs of the cyclohexane solutions of the core (left), the core@shell particles (middle) and aqueous solution of UPG (right) taken in the dark under excitation of a 980 nm laser beam. **b** T_1_-MRI maps & values, and **c** plots of R_1_ versus Gd^3+^ concentrations for UPGs in aqueous solution. The corresponding relaxivity value of UPGs is estimated to be r_1_ = 3.84/mM/s

Previous studies have demonstrated that the core@shell structures are more effective for enhancing upconversion luminescence intensity of UCNPs via improving the passivation of photogenic dopants that encapsulates onto the nanoparticle surface [29]. To demonstrate the efficacy of the shell coating, emission spectra of the three nanoparticles were characterized under 980 nm laser excitation. As displayed in Fig. 4a, the luminescence intensity of the core@shell nanoparticles, normalized according to Y^3+^ concentration, was increased by a factor of 3.6 compared with that for the core nanoparticles, giving rise to very bright green light as shown by the full-color photographs of the UCNP and UCNP@Gd nanoparticles in cyclohexane and deionized water under 980 nm laser with 300 mW, respectively. Although the luminescence intensity of the UPG has somewhat weakened compared to the UCNP@Gd, it still benefits the in vivo detection sensitivity.

Gd^3+^-doped UCNPs has been demonstrated to be an attractive method for realizing both fluorescent and MR imaging of tumors [30]. Toward this demand, core@shell structured UCNP@Gd nanoparticles were prepared on the basis of previous literature. The surface-associated Gd^3+^ ions additionally endow the nanoparticles with contrast-enhancing effect for MR imaging. By linear regression fitting of the R_1_ values at different Gd^3+^ concentrations, as shown in Fig. 4b, c, the molar relaxivities, r_1_, of the UPG nanoparticles were determined to be 3.84/mM/s.

### Toxicology assays of the micelles in vitro and in vivo

**Fig. 5:**
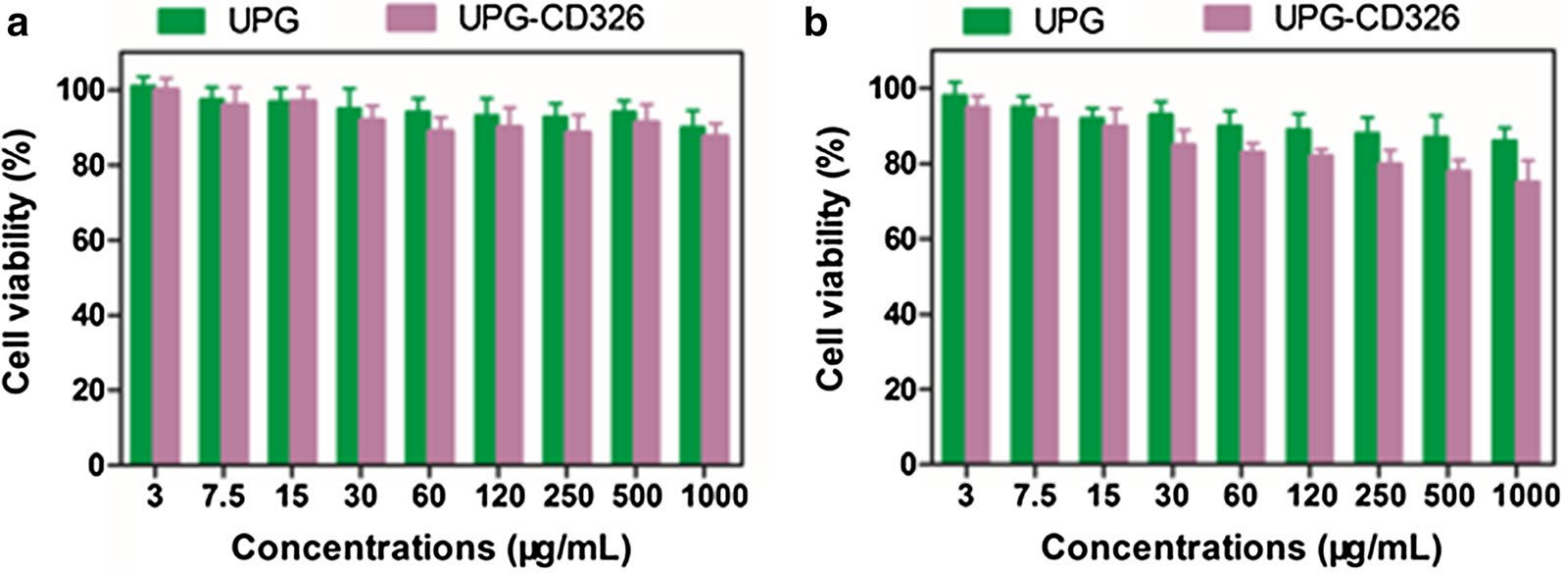
In vitro toxicity evaluation of RAW 264.7 macrophages after co-incubation with UPG/UPG-CD326 for (**a**) 24 and (**b**) 48 h by Cell Counting Kit-8 (CCK-8) assay

Before performing the in vitro biological experiments, the cytotoxicity of UPG/UPG-CD236 micelles was firstly conducted against RAW 264.7 macrophages by using CCK-8 assay. After coincubation with different concentrations of the micelles for 24 h/48 h, as displayed in Fig. 5, the cell viability of RAW 264.7 exceed 80% even up to relatively high micelles concentrations of 1000 µg/mL for 24 or 48 h. One possible major reason might be that PEGlation of UCNPs endowed a biosafe monolayer preventing the release of toxic ions, and minimizing the potential toxicity. Obviously, the negligible cytotoxicity of the micelles demonstrates their superior in vitro biocompatibility, which paves the way for their following dual-modality imaging.

**Fig. 6:**
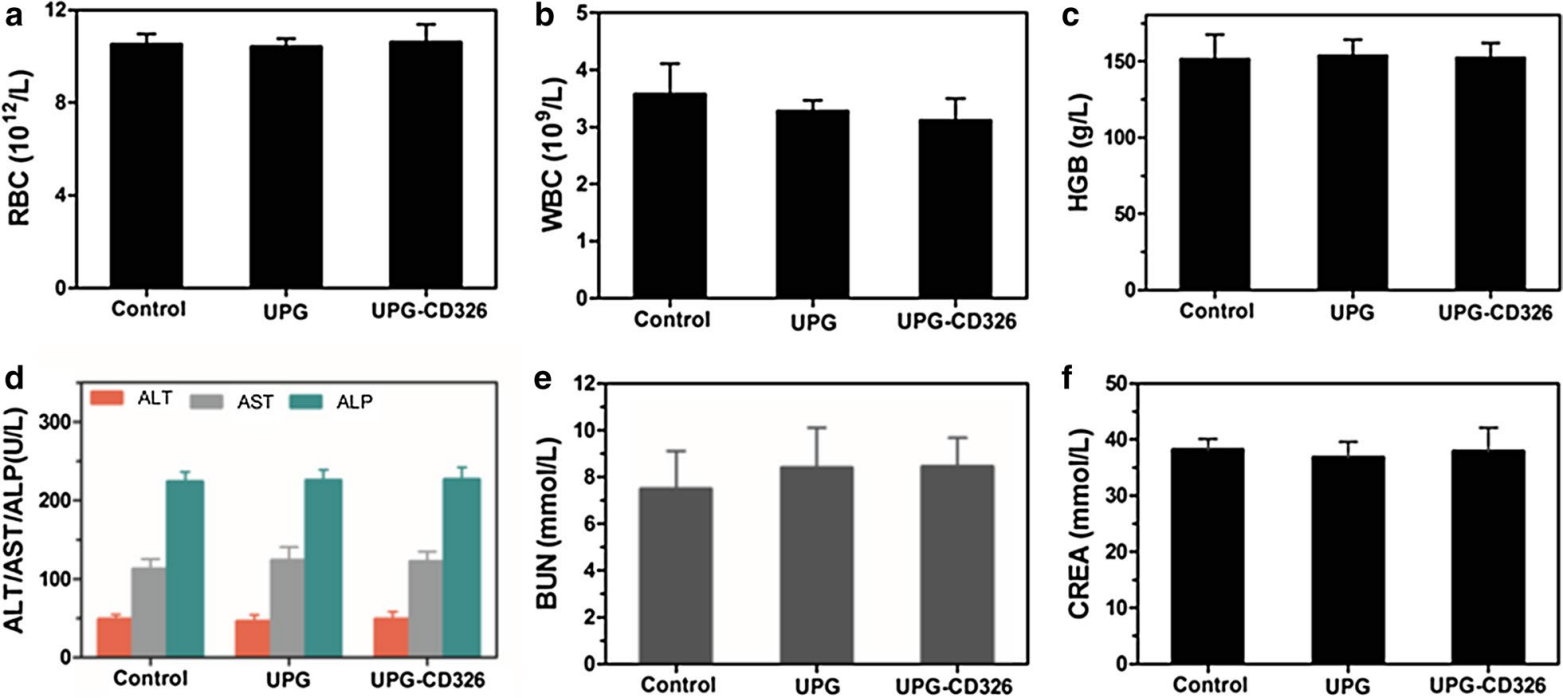
In vivo toxicology study and serum biochemistry results obtained from balb/c nude mice after 7 days postintravenous injection with 150 µL physiological saline of UPG/UPG-CD326 (25 mg/mL): Complete blood panel markers including: **a** red blood cells (RBC), **b** white blood cells (WBC), **c** hemoglobin (HGB); liver function markers including: **d** alanine aminotransferase (ALT), aspartate aminotransferase (AST), and alkaline phosphatase (ALP); kidney function markers including: **e** blood urea nitrogen (BUN), and **f** creatinine (CREA); untreated healthy mice were used as the control. Statistics were based on six mice per data point

**Fig. 7:**
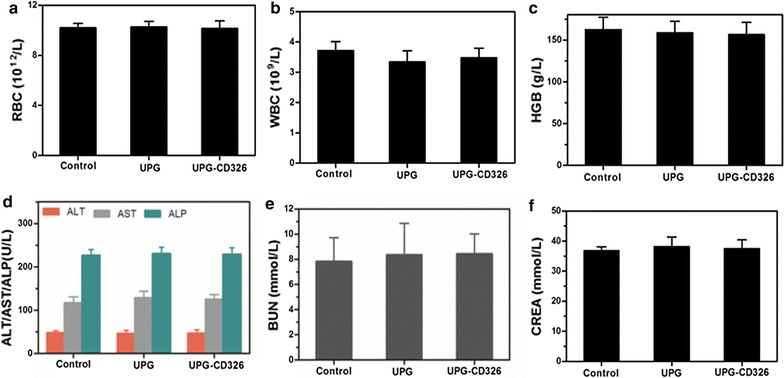
In vivo toxicology study and serum biochemistry results obtained from balb/c nude mice after 35 days postintravenous injection with 150 µL physiological saline of UPG/UPG-CD326 (25 mg/mL): Complete blood panel markers including: **a** red blood cells (RBC), **b** white blood cells (WBC), **c** hemoglobin (HGB); liver function markers including: **d** alanine aminotransferase (ALT), aspartate aminotransferase (AST), and alkaline phosphatase (ALP); kidney function markers including: **e** blood urea nitrogen (BUN), and **f** creatinine (CREA); untreated healthy mice were used as the control. Statistics were based on six mice per data point

Considering the following imaging study in vivo, and encouraged by the above cytotoxicity results, we then investigated in vivo toxicology experiments. Firstly, the long-term toxicology of the micelles in vivo was investigated by blood and biochemical examinations. As displayed in Fig. 6, RBC, WBC, HGB, liver and kidney function markers were within the normal ranges after 7 days post-intravenous injection with 150 µL physiological saline of UPG/UPG-CD326 (25 mg/mL). Similar results were shown from Fig. 7, all of the data were within normal limits even 5 weeks post-injection, which reconfirmed that the micelles did not cause any obvious changes in all of the examined parameters. In addition, no abnormal behaviors were observed among the mice groups treated with two types of the micelles, respectively. These results convincingly evidence that the synthesized UPG/UPG-CD236 micelles are biocompatible in vivo.

### Cellular uptake study using confocal laser scanning microscopy

**Fig. 8:**
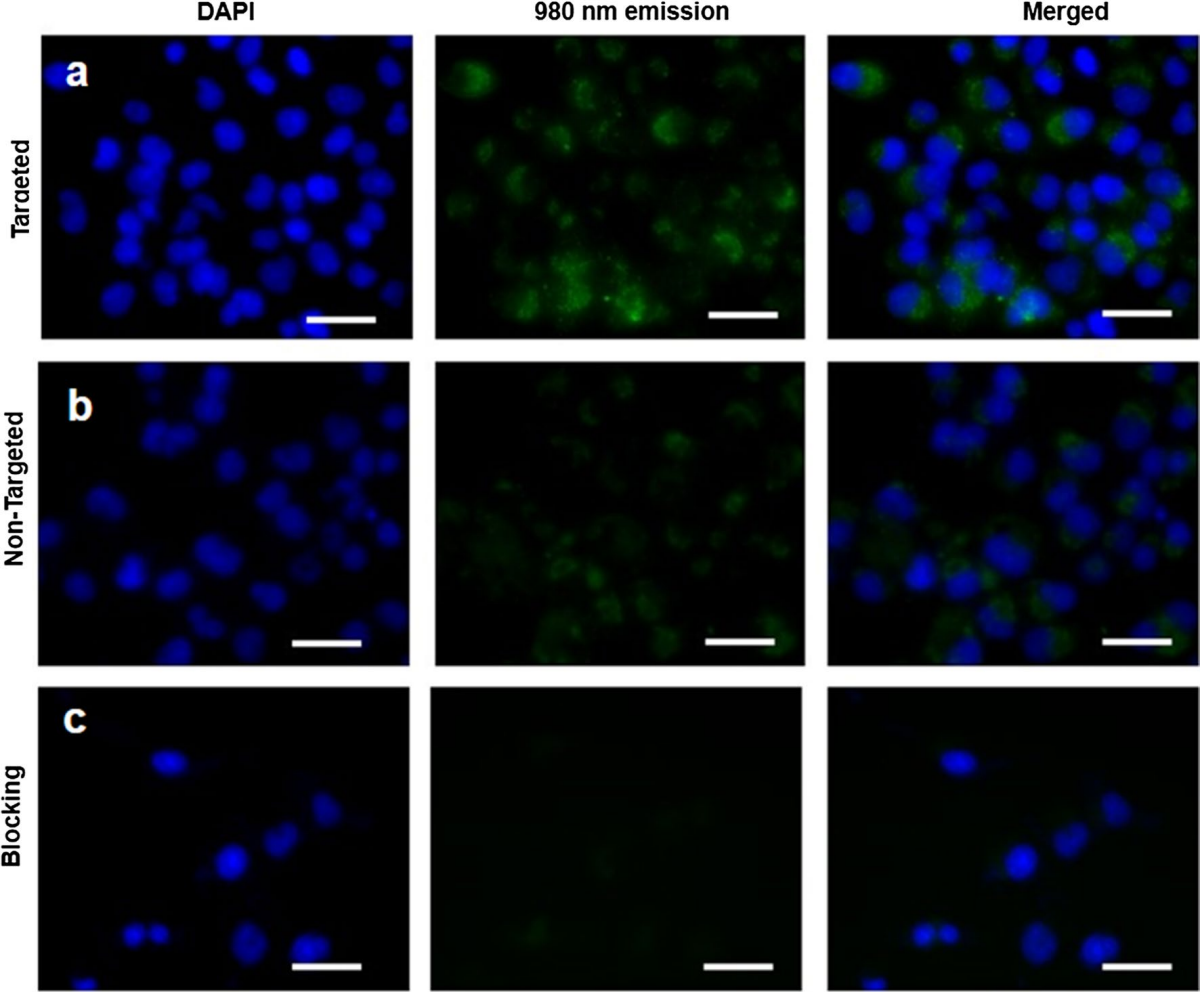
In vitro cellular uptake study of the UCNP-based micelles in BxPc-3 cells. Cells were treated with (**a**) targeted (i.e., UPG-CD326) micelles, and **b** nontargeted (i.e., UPG) micelles (200 μg/mL), as well as (**c**) the combination of free anti-human CD326 (3 × 10^−6^ M) and targeted micelles (i.e., blocking) for 2 h at 37 °C. Blue luminescence is from the nucleus after being stained with DAPI. Upon 980 nm irradiation, the UPGs micelles emit green luminescence from the cytoplasm of the cells treated with UPG-CD326 and UPG micelles

The upconversion luminescence emission of UCNPs under 980 nm excitation was utilized for micelle detection. CD326 are overexpressed onto the membrane of BxPc-3 cells, and CD326 is known as one biomarker of pancreatic cancer cells. Thus, utilizing CD326 as one potential target for realizing optical imaging is of great significance. Subsequently, BxPc-3 cells were incubated with 200 μg/mL DMEM solutions of UPG/UPG-CD326 for 2 h and washed three times with PBS before fluorescence imaging. Subsequently, we performed the cellular uptake assay of UPG/UPG-CD236 with a CLSM equipped with a CW 980 nm source. As shown from Fig. 8, a higher green intensity was observed clearly in the cells treated with targeted micelles compared to those treated with nontargeted micelles, indicating that anti-human CD326 can effectively enhance the cellular uptake of micelles through an antigen–antibody-mediated endocytosis process. On the contrary, in the blocking experiment, when CD326 on the BxPc-3 cells were saturated by free anti-human CD326 antibodies, the extent of cellular uptake of targeted micelles was similar to that of the nontargeted micelles. Taken together, these results confirm the ability of UCNP-based micelles for bioimaging, as well as the excellent CD326-targeting ability of the anti-human CD326 antibody.

### UCL imaging in vivo

**Fig. 9:**
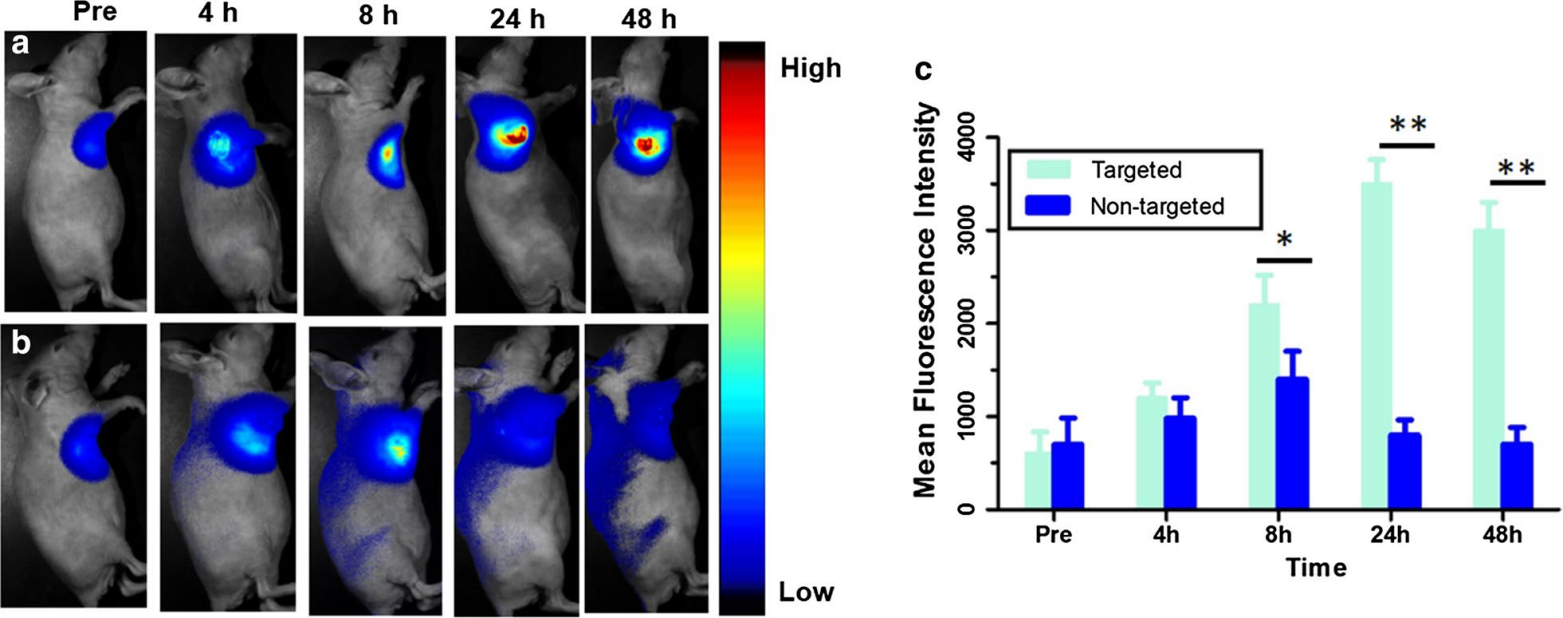
In vivo upconversion luminescence (UCL) images obtained before and at different time points after intravenous injection of the targeted (i.e., UPG-CD326) (**a**) and nontargeted (i.e., UPG) (**b**) micelles. **c** Comparison of the mean fluorescence intensities (MFI) between targeted and nontargeted groups at the different time points (*p < 0.05, **p < 0.01)

As aforementioned, UCNPs emit the upconversion luminescence under 980 nm light irradiation, which was employed for as an optical imaging probe in vivo [31]. The UCL images were gained at the designated time points including pre-injection, 4, 8, 24 and 48 h post-injection of the two micelles through tail vein. As shown in Fig. 9a, b, green luminescence was clearly visible in both the non-targeted group and targeted group under 980 nm irradiation. Moreover, a much stronger signal was captured in the mice treated with UPG-CD326 micelles, reiterating the excellent in vivo active targeting ability of anti-human CD326. In contrast to UPG micelles that only exhibit passive tumor-targeting ability via the EPR effect, UPG-CD326 micelles possessed both passive and active CD326 targeting abilities, thereby leading to a much higher accumulation of micelle in the tumor tissue even at the time point of 48 h. As shown in Fig. 9c, the average fluorescence intensities of the targeted group are statistically different at the time points of 8, 24 and 48 compared to those of the non-targeted group, respectively.

Although the targeting ability of UCNP-CD326 micelles may be impaired by the adsorption of nonspecific proteins in microenvironment [32], the efficient in vivo imaging of the xenograft tumor model demonstrated that if the nonspecific adsorption of plasma proteins would occur with respect to the micelles, it might be within a very narrow range or even negligible. This study has demonstrated that the micelles conjugated with anti-human CD326 as an active targeting ligand can effectively accumulated in the tissues of pancreatic cancer, which holds great promise for further studies in the future.

### MR imaging in vivo

**Fig. 10:**
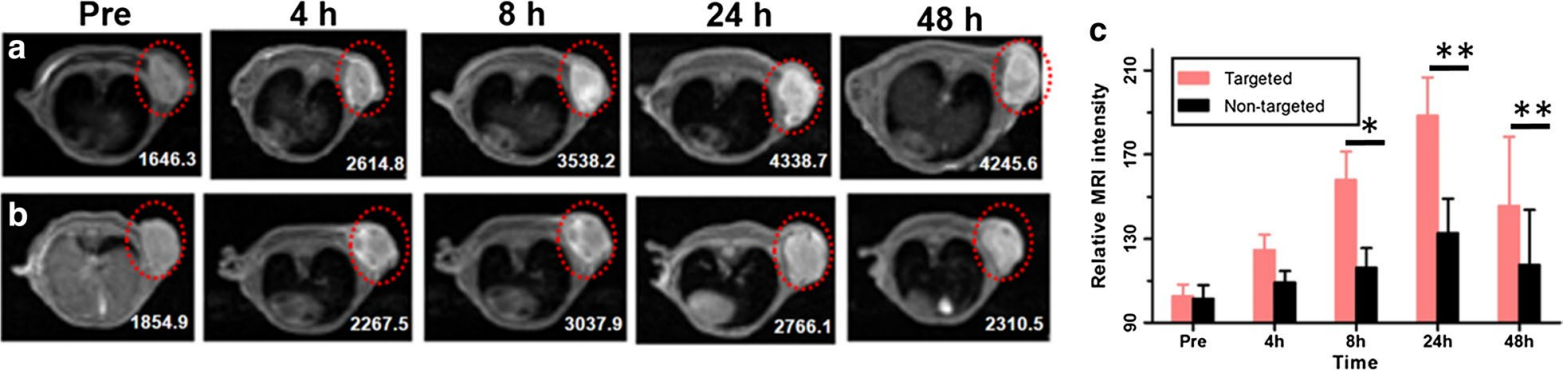
In vivo T_1_-MRI images of BxPc-3 tumor-bearing mice after intravenous injection of the targeted (UPG-CD326) (**a**) and nontargeted (UPG) (**b**) micelles at designated time points (Red dotted circles show the site of tumor, values mean T_1_, unit: ms). **c** Comparison of the relative MRI intensities between targeted and nontargeted groups at the different time points (*p < 0.05, **p < 0.01)

To further verify the active targeting ability of UPG-CD326, tumor-bearing mice were conducted to MR imaging experiments after intravenous injection of the UPG/UPG–CD236 micelles. As shown in Fig. 10a, b, the drastically increased relative T_1_ value of the targeting group at 8 h post-injection in comparison to the non-targeting group demonstrated the targeting ability of the antibody conjugated micelle to the implanted xenograft tumor in vivo. More excitingly, the relative T_1_ value of the non-targeted group was gradually decreased to the baseline of pre-injection, while the value remained the same as that of 8 h in the targeted group. This may be attributed to more accumulation of UPG-CD236 in the tissue of tumor due to the conjugation of anti-human CD326. Similarly, as seen in Fig. 10c, the relative MRI intensities of the targeted group were statistically different compared to those of the nontargeted group at the time points of 8, 24 and 48 h, which further confirmed a greater tumor uptake of the targeted micelles compared to the nontargeted ones. Thus, this unique UPG-CD326 micelle may evolve as a promising MR imaging probe for active targeting pancreatic cancer.

### Biodistribution and targeting efficiency of the micelles

**Fig. 11:**
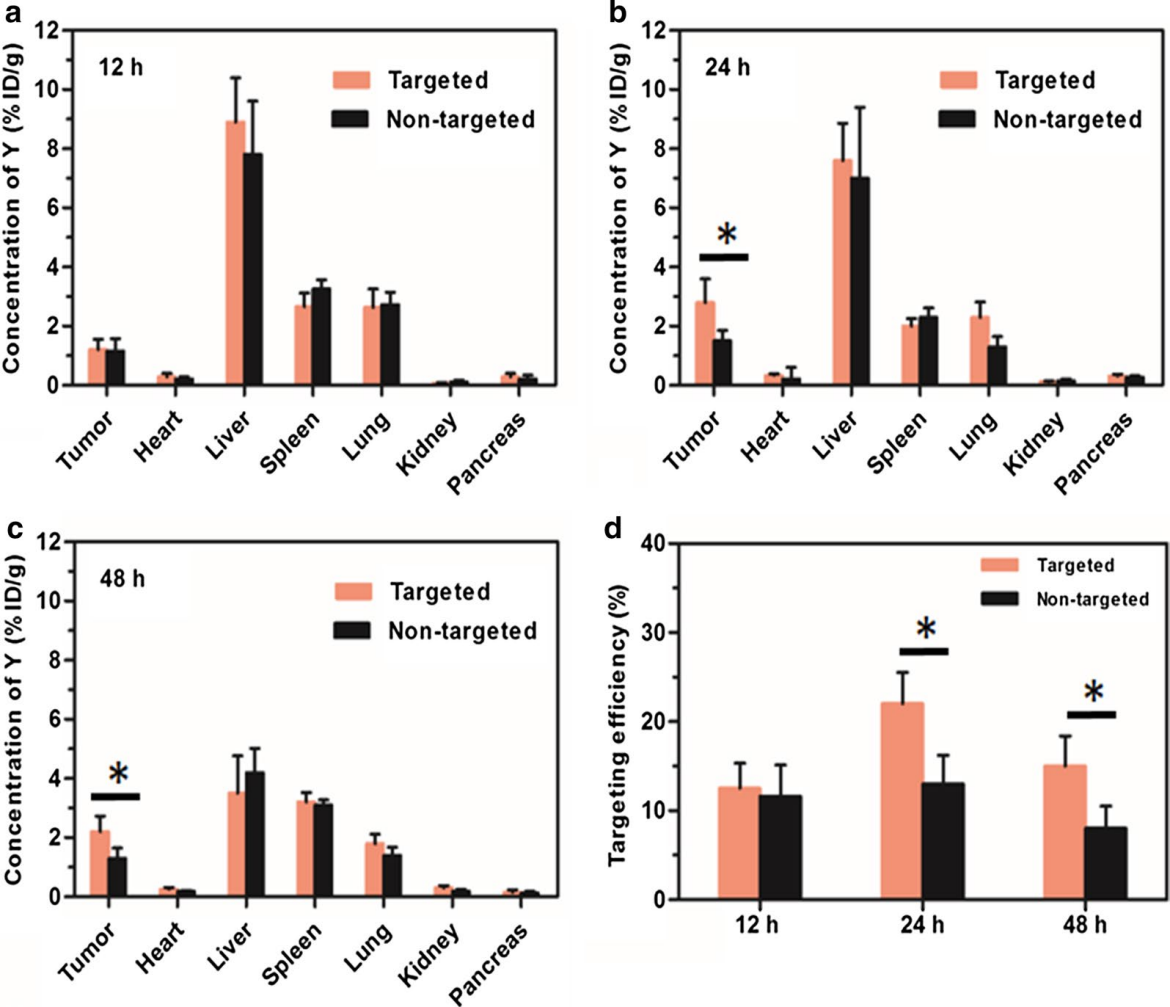
Biodistribution of UPG-CD326 and UPG micelles in mice post-injection. The concentration of Y element in the tumor and tissue samples was evaluated by ICP-MS at (**a**) 12, (**b**) 24 h, and (**c**) 48 h post-injection. **d** Targeting efficiency of UPG-CD326 and UPG micelles in mice post-injection. Data represent mean ± SD of 5 replicate mice at each sacrifice time. *p < 0.05

For quantitative measurement of in vivo distributions and targeting efficiency of UPG-CD326 and UPG micelles, the levels of Y element in collected tissue samples (5 mice/group) were measured at different time points via ICP-MS. As displayed in Fig. 11, the micelles mainly accumulated in the liver and spleen at 12 h post-injection, demonstrating that the synthesized micelles could accumulate in the organs of the reticuloendothelial system due to EPR effect. At 24 h post-injection, the accumulation amount of the UPG-CD326 micelles within tumors gradually increased and reached a peak, which was significantly higher than that of UPG micelles (*p* < 0.05). This statistically difference was thoroughly due to active targeting ability of UPG-CD326 micelles. However, the Y content dropped in the tumors and major organs at 48 h post-injection, and this is presumably due to slow clearance of the nanoparticles through a hepatobiliary route. Besides, the targeting efficiency of UPG-CD326 and UPG micelles are statistically different at 24 and 48 h post-injection, respectively. Obviously, this results reconfirmed that anti-human CD326 conjugated on the micelles had good active targeting ability.

### Cellular localization of the micelles by Bio-TEM observation

**Fig. 12:**
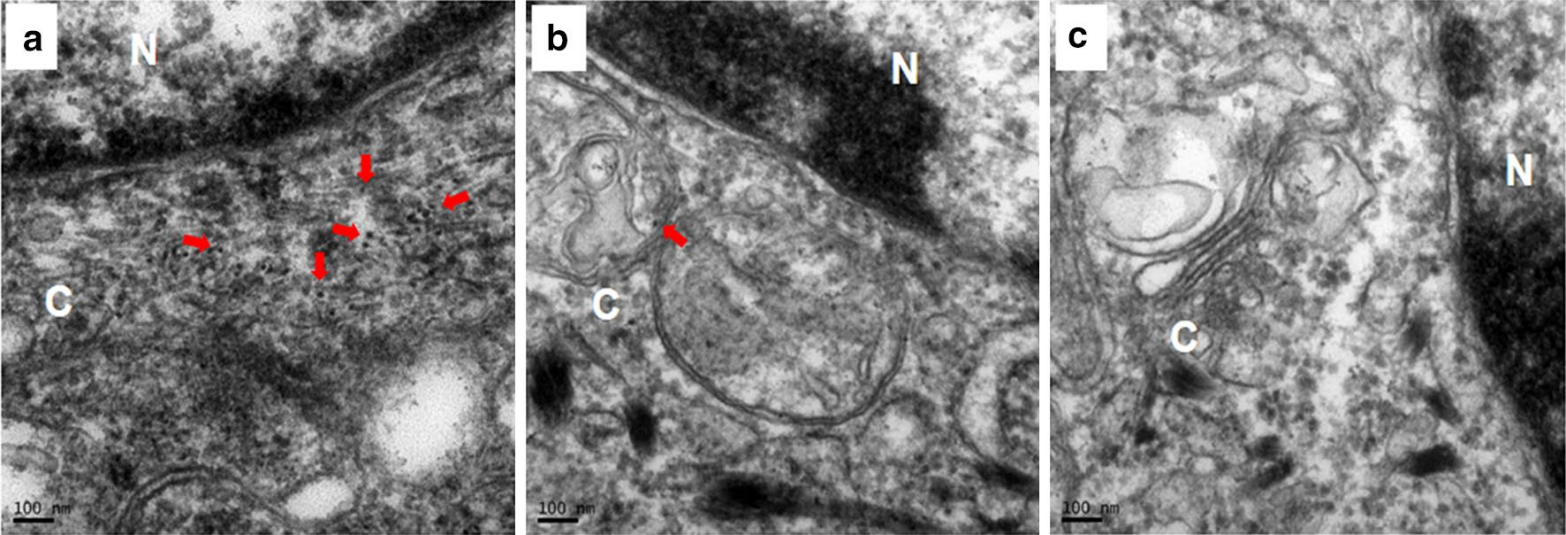
Bio-TEM images of pancreatic cancer tissues from BxPc-3-tumor bearing mice after 48 h post-injection with (**a**) UPG-CD326 micelles, (**b**) UPG micelles and (**c**) normal saline. Red arrow show the UPG micelles reside around the nucleus. C and N mean cytoplasm and nucleus, respectively

As shown in Fig. 12, more UPG-CD326 micelles were observed around the nucleus in the tumor tissues from the mice treated with targeted micelles, indirectly reconfirming the superior in vivo targeting ability of the anti-CD326 ligand. In contrast to nontargeted micelles that only own passive targeting ability via the EPR effect, anti-CD326-grafted micelles owned both passive and active targeting abilities, thereby resulting in a remarkably higher accumulation of the micelles in the tumor tissues. All the above Bio-TEM results confirm that with the conjugation of anti-CD326 ligand, the efficient intracellular localization of the UPG-CD326 micelles has been achieved.

## Conclusions

In summary, CD326-targeted UCNP-based micelles were constructed for simultaneous dual-modality UCL/MRI imaging. In vivo UCL/MR imaging experiments have demonstrated the satisfying ability of the targeted micelle for detecting pancreatic cancer, owing to the excellent tumor-targeting specificity and imaging sensitivity enabled by the rational micelle design. The CD326 targeting antibody was capable of remarkably increasing the cellular and tumoral uptake of the micelles, without any apparent systemic toxicity. Therefore, the current studies may pave a highly effective way for the diagnosis as well as provide a powerful tool for enhancing the prognosis of pancreatic cancer patients.

## Abbreviations

UCNPs: upconversion nanoparticles
Gd^3+^: gadolinium ion
PEG: polyethylene glycol
EPR: enhanced permeability and retention
EpCAM: epithelial cell adhesion molecule
UPG: UCNP-based micelle
MR: magnetic resonance
YCl_3_·6H_2_O: yttrium(III) chloride hexahydrate
YbCl_3_·6H_2_O: ytterbium(III) chloride hexahydrate
ErCl_3_·6H_2_O: erbium(III) chloride hexahydrate
GdCl_3_·6H_2_O: gadoliniumchloride hexahydrate
NH_4_F: ammonium fluoride
OA: oleic acid
ODE: 1-octadecene
DAPI: 4,6-diamidino-2-phenylindole dihydrochloride
NaOH: sodium hydroxide
MPEG-2000-DSPE: *N*-(carbonyl-methoxypolyethleneglycol-2000)-1,2-disteroyl-sn-glycero-3-phosphoethanolamine
DSPE-PEG2000-Mal: 1,2-distearoyl-sn-glycero-3-phosphoethanolamine-*N*-[maleimide(polyethylene glycol)-2000
CCK-8: Cell Counting Kit-8
UCNP@Gd: NaYF_4_:Yb,Er@NaGdF_4_
UPG-CD326: UCNP@Gd@PEG-CD326
TEM: transmission electron microscopy
ICP-MS: inductively coupled plasma mass spectrometry
NIR: near infrared
FTIR: Fourier transform infrared
DLS: dynamic light scattering
TR: repetition time
TE: echo time
R_1_: relaxivity coefficient
CLSM: confocal laser scanning microscopy
XRD: X-ray powder diffraction
EDX: energy-dispersive X-ray
RBC: red blood cells
WBC: white blood cells
HGB: hemoglobin
ALT: alanine aminotransferase
AST: aspartate aminotransferase
ALP: alkaline phosphatase
BUN: blood urea nitrogen
CREA: creatinine
